# Drug Utilization Pattern in Patients with Different Types of Dementia in Western India

**DOI:** 10.1155/2014/435202

**Published:** 2014-08-27

**Authors:** Mansi Patel, Anuradha Joshi, Jalpa Suthar, Soaham Desai

**Affiliations:** ^1^Department of Pharmacology, Ramanbhai Patel College Of Pharmacy, Charotar University of Science and Technology, Changa, Anand, Gujarat 388421, India; ^2^Department of Pharmacology, Pramukhswami Medical College, HM Patel Centre Medical Care & Education, Gokalnagar, Karamsad, Anand, Gujarat 388325, India; ^3^Department of Neurology, Pramukhswami Medical College, Karamsad, Anand, Gujarat 388325, India

## Abstract

*Background.* Dementia is one of the most frequent disorders among elderly patients, reaching to epidemic proportions with an estimated 4.6 million new cases globally annually. Partially effective treatments are available for dementia. *Aims & Objectives.* We aim to study drugs used in dementia and find out frequency of types of Dementia. *Method.* This was an observational study conducted at rurally based tertiary care hospital. Prospective data was collected from outpatient department, while retrospective data was collected from medical records. Descriptive statistics were used to analyze data. *Result.* Total 125 prescriptions of patients diagnosed with dementia were analyzed. Alzheimer's dementia was most common (65.6%), followed by vascular dementia (21.6%), and frontotemporal dementia (10.4%), with the rarest being Lewy body dementia in (2.4%) cases. 60.57% of patients were males. Mini Mental Score Examination mean score was 15.93 ± 1.37. Frontal Battery Assessment mean score was 4.75 ± 1.01. Prescribed drugs were Donepezil (68.49%), Rivastigmine (13.63%), Donepezil + Memantine (6.43%) and Galantamine (12.83%), Quetiapine (38.46%), Lorazepam (23.07%), Clozapine (11.53%), Escitalopram (10.25%), Haloperidol (3.84%), Zolpidem, Sertraline, Olanzepine (2.56%), Nitrazepine, Lamotrigine, Fluoxetine, Tianeptine (1.28%), Folic acid, and Vitamin B12, respectively. *Conclusion.* Alzheimer's is the most common type of dementia while Donepezil was the most frequent drug.

## 1. Introduction

Dementia is one of the most disabling disorders afflicting adult and elderly population reaching to epidemic proportions with an estimated 4.6 million new cases worldwide each year [1]. It is expected that the burden of dementia will be increasing in developing countries due to increase in longevity and increasing prevalence of risk factors such as hypertension and stroke [2, 3]. Looking into the Indian scenario, in 2005 it was estimated that 24.3 million worldwide and 1.8 million people in India are affected with dementia [4]. Moreover the Delphi consensus study estimated its prevalence to be 1.9% in people above 60 years of age in India [5] as well as South Asia, with an annual incidence of 4.3/1000. Hence the disease has gained importance in recent times. With increasing life expectancy, the prevalence of dementia will increase dramatically in the next few decades, and will have a staggering emotional and economic impact. Thus, dementia and cognitive impairment are one of the major public health concerns in the 21st century.

Dementia is characterized by progressive loss of memory or mental faculties such as language, judgment planning leading to impairment of daily activities, and deficiency in social interaction. While the treatment of dementia is basically directed towards management of cognitive and behavioral symptoms of dementia, partially effective treatments are available for most core symptoms of dementia. The treatment of dementia varies through the course of its illness, because symptoms evolve over time. Various drugs have been utilized for delaying cognitive decline as well as improving the behavioural symptoms associated with dementia. Acetyl cholinesterase inhibitors including Donepezil, Rivastigmine, and Galantamine have been recommended to be used in mild to moderate cases of dementia while Memantine is the only drug recommended for severe cases of dementia. Looking into the morbidity, appropriate utilization of antidementia therapy is highly essential for these patients. On the other hand psychosis, agitation and other behavioral symptoms are reported in patients with dementia causing lot of distress in caregivers. The atypical antipsychotics like Olanzepine, Quetiapine, Risperidone, and Clozapine have become the first line pharmacological treatments for behavioral symptoms in dementia patients. However the increased risk of mortality reported with the use of atypical antipsychotics has resulted in considerable skepticism over their use in the dementia patients.

Published Indian literature [6] on various drugs used in dementia in India as well as in Gujarat (a part of western India) is still in its infancy. The prescription pattern of antidementia drugs and use of antipsychotics, vitamin B12, and folic acid has still not much been studied in developing countries.

Drug utilization studies derives its importance in Pharmacoepidemiology from the fact that it is a part of medical audit involving the monitoring and evaluation of various prescriptions of Medical Practitioners to ensure rationality in medical care. In this article, we highlight the present Indian scenario of dementia in relation to its epidemiology, comorbidities and drug utilization in this particular region. Adjuvant supplements of vitamin B12 and folic acid are also being administered by clinicians to improve cognitive function of people. According to consensus guidelines on treatment of dementia before selecting a supplementation strategy, it is recommended that clear goals and target populations be set and checking of vitamin B12 and folic acid levels should be included in the diagnostic workup in patients undergoing assessment for dementia [7].

In light of this in current practice there seems to be scarcity of data on prior determination of serum vitamin B12 and folic acid status by clinicians before supplementing these vitamins in patients of dementia. Overuse of such multivitamins and folic acid, along with antidementia medications, without checking the serum levels may lead to an overprescribing of these drugs as well as increased economic burden to the patients.

Thus, this study was undertaken in patients presenting with impaired cognition, diagnosed to be suffering from dementia, to provide a glimpse of the drug utilization pattern amongst patients with dementia in a rural based medical teaching hospital in western India.

## 2. Materials and Methods

The study reported here was carried out at a rurally based tertiary care hospital (ShriKrishna Hospital, H.M.Patel Centre Medical Care & Education, Gokalnagar, Karamsad, Gujarat.) in western India. This was a cross sectional, point prevalence, open observational study, carried for a period of six months (July 2013 to December 2013). The study was initiated after the approval from the Ethics Committee of the hospital for the same (Approval No. HREC/FCT/46/APPROVAL/21).

Prospective as well as Retrospective data was collected. Prospective data was collected from outpatient department of hospital from July 2013 to December 2013. The prospective data was collected from the outpatient departments of the neurology, medicine, and psychiatry of the hospital from the prescriptions written by the respective medical professionals, that is, Neurologists (MBBS, MBBS, MD (Internal Medicine), DM (Neurology) [All India Institute of Medical Sciences] Consultant Neurologist), Physicians (MBBS, MD Internal Medicine) and Psychiatrist, (MBBS, MD Psychiatry) after obtaining the informed consent of the patient, while Retrospective data was collected from medical record section, that is, data was obtained from preserved past medication files of the patients visiting the hospital between the years 2008 to June 2013 after seeking permission for the same.

The study criterion included all patients who were diagnosed with dementia on the basis of DSM IV classification of disorders criteria. In addition, all patients also underwent MMSE (Mini Mental State Examination) and FAB (Frontal Assessment Battery) evaluations used for assessing the patients with dementia. MMSE is a brief, structured mental status examination for global cognitive function. The FAB is a brief tool that can be used at the bedside or in a clinic setting to assist in discriminating between dementias mainly between frontotemporal dementia and dementia of Alzheimer's Type. All the obtained data was entered into a predesigned case record form. Case record form included all the information of patient like demographic details, their education level, socioeconomic class, details of MMSE and FAB scores, and drugs prescribed in dementia as well as various comorbid conditions. All the prescriptions were analyzed to find out the sample characteristics, frequency of different types of dementia, and prescribing pattern in these patients mainly focusing on the antidementia, antipsychotics, and vitamin supplementation.

### 2.1. Statistical Analysis

The data was suitably coded and compiled on a Microsoft Excel sheet and descriptive statistics was used to analyze the data.

## 3. Results

### 3.1. Demographic

**Figure 1:**
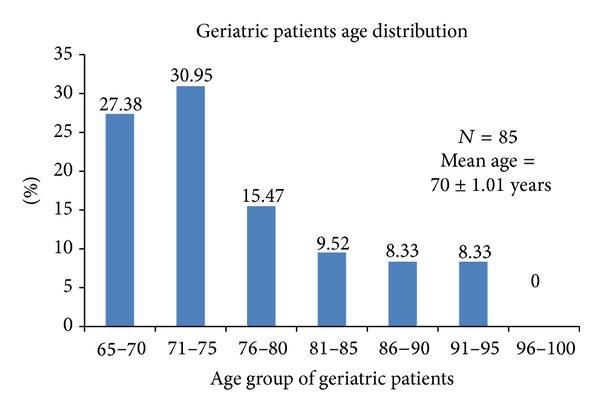
Geriatric population age distribution of dementia patient.

A total of 125 prescriptions were analyzed. Looking into the demographics of the patients, mean age of the population was found to be 71 ± 1.51 years (72 ± 2.01 in Prospective data and 70 ± 1.01 in Retrospective data) which are depicted in Table 1. A total of 85 out of 125 patients were found to belonging to the geriatric category (70.20%) as depicted in Figure 1.

Regarding Gender distribution, males dominated the population of study that is, 60.57% (63.1% in Prospective data while 58.61% in Retrospective data) while females 39.43% (36.82% in Prospective data while 41.32% in Retrospective data) as depicted in Table 1.

Socioeconomic class of the patients depicted that most of them belonged to the lower socioeconomic class, that is, 59.2%. Regarding education of patients in the sample, it was also noted that almost 8.0% of the patients were illiterate. A total of 56.0% of the patients were high school graduates. The percentage of population who were graduates was 24.0% while postgraduates were very less 12.0%, as shown in Tables 2 and 3.

### 3.2. MMSE and FAB Score

**Figure 2:**
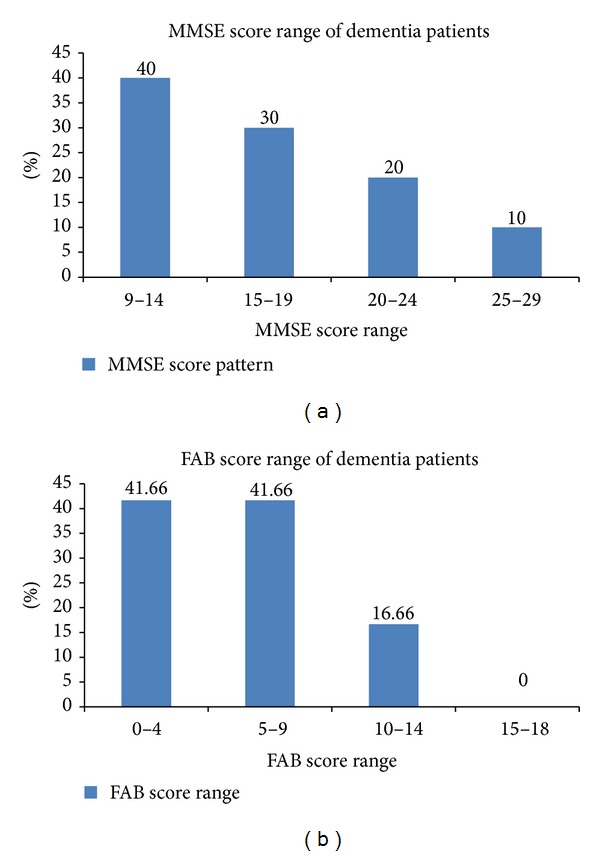
(a) depicts the MMSE score pattern of dementia cases. (b) depicts FAB score pattern of dementia cases.

Mini Mental State Examination (MMSE) and Frontal Assessment Battery (FAB) score was conducted to establish the diagnosis of the stage of dementia, to determine the advancement of diseases in dementia patients, and also to predict the mental capacity of patient. In our study the MMSE score ranges from 9–29 out of 30. A total of 40% of the population had a score ranging within 9–14. Approximately 30% of the population had a score within 15–19. The remaining 30% had a score ranging within 20–30. This is shown in Figure 2(a) The FAB scores ranged between 2 and 14 out of 18. Approximately 80% of the population had a score between the ranges of 0 and 9. Rest of the population had a FAB score ranging within 10–14. None of the patient had a score greater than 14. This is shown in Figure 2(b). Mini Mental Scale Examination mean score was 15.93 ± 1.37 while Frontal Assessment Battery mean score was 4.75 ± 1.01.

### 3.3. Subtypes of Dementia

Out of 125 patients, the commonest type of dementia reported was Alzheimer's disease found in 65.6% of the cases, (82 patients, Male = 45, Females = 37, Mean age = 72 ± 1.2 years) followed by vascular dementia which was found in 21.6% of the cases (27 patients, Male = 13, Female = 14, Mean Age = 70 ± 0.1 years) and followed by Frontotemporal dementia in 10.4% cases (13 patients Male = 9, Female = 4, Mean Age = 63 ± 0.7 years), with the least common subtype being Lewy body dementia found only in 2.4% cases, (3 patients, Males = 2, Females = 1, Mean Age 73 ± 0.4 years) as depicted in the Table 4.

**Figure 3:**
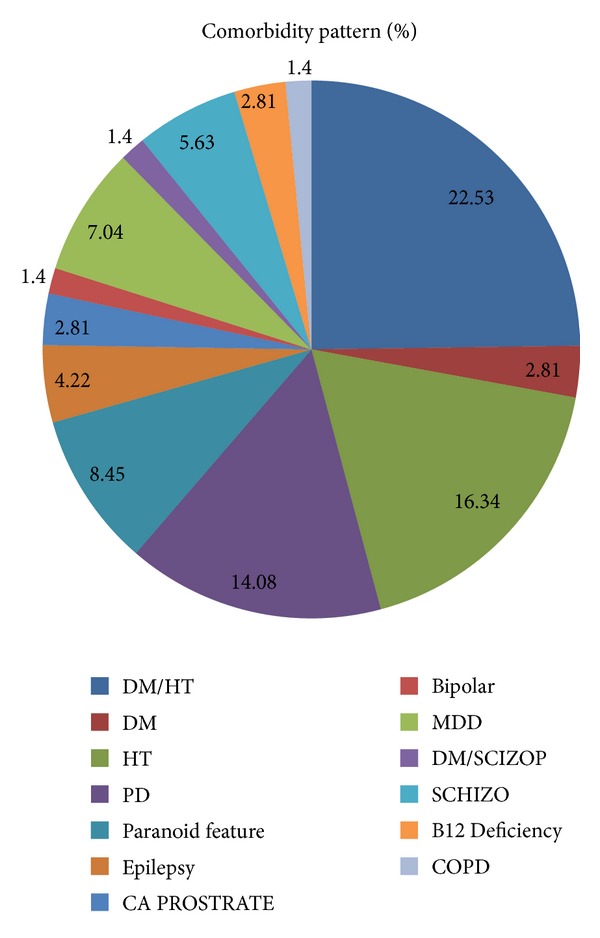
Representation of comorbidity distribution of dementia cases.

Similarly the distribution of different comorbidities associated with the dementia patients is represented in Figure 3. A total of 56.8% of the patient had one or more associated comorbidity. Regarding the co-morbidity pattern in dementia patients in the study, we found that hypertension and diabetes mellitus were found to be the most common type of associated comorbidity. However, these comorbidities were effectively managed with antihypertensive drugs and antidiabetic drugs.

### 3.4. Drug Utilization Pattern in Dementia Patients

Patients included in the study were those of who were being treated with the Anticholinestrase drugs like Donepezil, Rivastigmine, Galantamine, Memantine either singly or in combination, and patients who were on concurrent medications like antipsychotics and vitamin B12 and folic acid therapy along with antidementia drugs.

**Figure 4:**
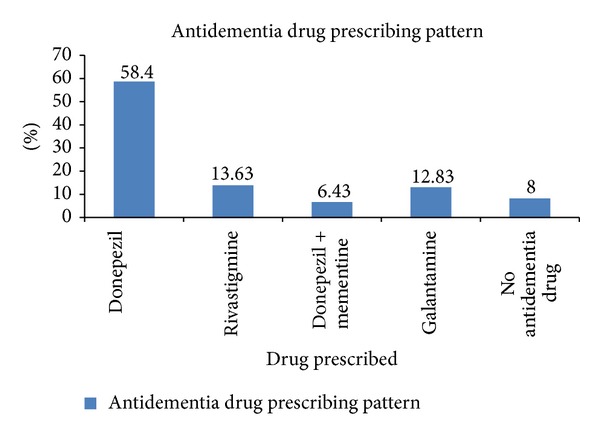
Depiction of antidementia drug prescribing pattern.

In the given study, a total of 125 patients suffering from dementia were included. Prescription analysis of dementia patients depicts that most popular drug of choice for the given condition was Donepezil (68.49%) in a dose range of 5 to 10 mg, Rivastigmine (13.63%) in a dose range of 1.5 to 3 mg, Donepezil + Memantine (6.43%) and Galantamine (12.83%), respectively, as shown in Figure 4. The doses of drugs used were 5 mg dose in 68.49% of cases and 10 mg dose in 31.50% of cases in Donepezil, while for Rivastigmine it was 1.5 mg dose in 41.17% of cases and 3 mg dose in 58.82% of cases. Memantine in combination with Donepezil 5 mg was prescribed in 6.43% cases and Galantamine 8 mg in 12.83% cases, respectively, as shown in Table 5.

**Figure 5:**
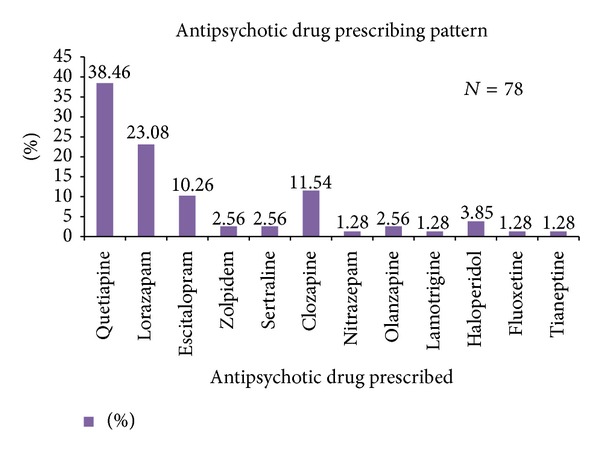
Depiction of antipsychotic drug prescribing pattern in dementia cases.

Along with antidementia drugs a large number of antipsychotics were prescribed to the dementia patients. Viewing the prescribing pattern, it was revealed that out of 125 patients 78 patients were prescribed at least one anti-psychotic drug. Among this commonest Antipsychotics used were atypical in nature that is, Quetiapine used in 30 (38.46%) cases, Clozapine in 9 (11.53%) cases, Olanzepine in 2 (2.56%) of cases, Older antipsychotic like Haloperidol in 3 (3.84%) of cases, Benzodiazepines like Lorazepam in 18 (23.07%) of cases, Nitrazepam in 1 (1.28%) of the cases, Non Benzodiazepine hypnotics like Zolpidem in 2 (2.56%) cases, Antidepressants like SSRI (Selective Serotonin Reuptake inhibitors) Escitalopram in 8 (10.25%) cases, Fluoxetine in 1 (1.28%) of the case, Sertraline in 2 (2.56%) of cases, Atypical Antidepressants like Tianeptine in 1 (1.28%) of the case while Antiepileptics like Lamotrigine in 1 (1.28%) of the case, respectively, as shown in Figure 5.

On an average in our study in geriatric group population it was noted that 0.72 antipsychotic drug per prescription was prescribed.

Detailed analysis of the prescriptions under study reveals that there was high prescribing of folic acid and vitamin B12 in the patients without performing test to find out the need for the same in patient. folic acid was prescribed in 69 cases out of 125 (55.2%) while vitamin B12 alone was prescribed in 77 cases (61.6%), folic Acid and vitamin B12 was concurrently prescribed in 46 cases out of 125 (36.8%).

On an average in the study each prescription had 4 ± 0.1 drugs prescribed in the retrospective population. On the other hand 5 ± 0.2 drugs were found to be prescribed in the prospective population.

## 4. Discussion

Dementia is a silent epidemic well observed in elderly throughout the world as well as in developing countries like India but they are poorly recognized and treated in developing countries including India [5]. The disease stands to be equally important since there are an estimated 35.6 million people with dementia worldwide. More than half of these individuals have Alzheimer's disease (AD) [4, 8–10] resulting in a progressive decline in cognition, function and communication, together with the frequent occurrence of neuropsychiatric symptoms. As mentioned earlier the published Indian literature is scarce. The prescription pattern of drugs used in dementia patients has not been much studied in developing countries where cost may be the most important factor in determining their choice. Henceforth in this study, we highlight the present Indian scenario of dementia in relation to its epidemiology, comorbidities, and drug utilization in this particular region.

Our study reports the mean age of patients to be 72 ± 2.01, 70.02% of the population in our study was found to be geriatric population and males dominated the study population (60.57%). In one of the similar study conducted at multiple centers by Gil-Néciga and Gobartt, it was found that the mean age of patients was 77 ± 6.6 years and that female dominated the study population [11]. On contrary, study by Jeschke et al. during the 5-year study period (2004–2008), in 577 patients with dementia highlights that the median age of the patient was found to be 81 years (IQR: 74–87) and females dominated the study population that is, 69% female [12]. This difference may be observed because in India females are a bit neglected gender and there generally seems to be low awareness of this condition. Moreover another reason for low female population in our study could be that as compared to males, elderly females with dementia who are not working, who are residing at home are not given much importance and so not brought to hospitals for treatment. While another community based study conducted by Ruitenberg et al. reported that there is no gender differences in the incidence of dementia up to high age and that after 90 years of age the incidence of Alzheimer's disease is higher for women than for men [13]. As ours was a hospital based study we cannot compare the same.

However regarding Socioeconomic statusmost of them belonged to the lower socioeconomic class which can be one of the important risk factors responsible for dementia. A study by Qian et al. found that individuals with lower socioeconomic status come into memory clinic later when the disease has progressed to dementia, while higher socioeconomic status individuals present earlier when the disease is still in its mild cognitive impairment stage. They concluded that higher socioeconomic status is associated with better cognitive functioning and increased use of cognitive enhancers [14].

Mini Mental State Examination mean score was 15.93 ± 1.37 while Frontal Assesement Battery mean score was 4.75 ± 1.01. MMSE works out to be a good tool in the diagnosis of dementia. Study reported by Kukull et al. highlights that the conventional cutoff score of <24 shows a sensitivity of 0.63 and a specificity of 0.96 [15].

In the present study we found that amongst the types of Dementia Alzheimer's disease was the most common type in 65.6% of the cases, (Mean age = 72 ± 1.2 years) followed by, Vascular dementia which was found in 21.6% cases (Mean Age = 70 ± 0.1 years), followed by Frontotemporal dementia in 10.4% cases, (Mean Age = 63 ± 0.7 years) and the least common subtype being Lewy body dementia found only in 2.4% cases, (Mean Age 73 ± 0.4 years) of the cases.

A clinic-based study from South India also depicts similar results, in which Alzheimer's disease was found to be the commonest in 38.3% of the cases, vascular dementia in 25.4% of the cases, followed by frontotemporal dementia (FTD) in 18.7%, diffuse Lewy body disease (DLB) in 8.9%, and mixed dementia in 8.6% of the patients, respectively [16]. As stated earlier findings of a community based study conducted by Ruitenberg et al. report that after 90 years of age the incidence of Alzheimer's disease is higher for women than for men and that incidence of vascular dementia was reported to be higher in men than women in all age groups [13].

A total of 56.8% of the patient had one or more associated comorbidity. Regarding the comorbidity pattern in dementia patients in the study, we found that hypertension and diabetes mellitus were found to be the most common type of associated comorbidity. These comorbidities were effectively managed with antihypertensive drugs and antidiabetic drugs. Details of same were not a part of the current study. A study reported by Poblador-Plou et al. also found similar results, where the two most frequent comorbidities both for men and women with dementia were hypertension and diabetes. Other comorbidities significantly associated with dementia were Parkinson's disease, congestive heart failure, cerebrovascular disease, anemia, cardiac arrhythmia, chronic skin ulcers, osteoporosis, thyroid disease, retinal disorders, prostatic hypertrophy, insomnia and anxiety, and neurosis [17].

Present study reveals that Donepezil (58.4%) was found to be the most prescribed antidementia drug in patients with mild to moderate dementia. Apart from Donepezil, Rivastigmine (13.63%) was found to be the second most popular choice of drug for dementia patients, Galantamine was preferred for 12.4% of the population, and combination of choline-esterase inhibitor, that is, Donepezil and Memantine was used in 6.43%. An audit involving Indian geriatric population which was performed by Prasad et al. on clinical practice of medications in dementia, his analysis concluded that the commonest antidementia drug used and alone prescribed was Donepezil (52%), while Rivastigmine alone was used in a single patient, Memantine alone was prescribed in 18% of cases, Galantamine alone was used in three patients only, whereas, Rivastigmine in six patients. A total of 12% were not prescribed any anti-dementia drug [6]. In another study conducted by Rungsanpanya et al. wherein analysis of 91 prescriptions of Dementia patients, reported that Donepezil was the most frequently prescribed drug (70%), followed by Rivastigmine (22.5%) [18]. Similar observation was seen in the study performed by Mucha et al. [19] which reported that there were 3,177 patients included in the study, Donepezil was received by 62.8% of the patients (*n* = 1,994); 17.2% received Galantamine (*n* = 546) and 20.1% received rivastigmine (*n* = 637). Exploring the reasons for the high use of Donepezil as a drug of choice we find that both Acetylcholinestrase inhibitors and Memantine had significant effects on cognition. The single daily dosing, improvement in functional and psycho-behavioral outcome as well as cost of treatment may have influenced the choice of prescription as compare to other Cholinestrase inhibitors. Moreover the clinicians experience and acumen with these drugs may have also influenced prescriptions. Donepezil was favored mainly in most of mild to moderate cases of dementia while Memantine effect on functional impairment was better in more severe patients. Moreover the standard guidelines for the treatment of dementia suggest the same [20]

The 3 mg dose of Rivastigmine was more popular because generally patient were visiting in early stages, with mild to moderate dementia, most patients with severe dementia in India would be staying at their own homes with adequate care by children accepting the disease and trying alternative medicines as compared to allopathic leading to low frequency of visits in outpatient departments of respective units and this seems to be highlighted in our study. Hence Rivastigmine higher doses were not documented or not seen in the current study while most studies from the west would include patients under proper, adequate hospice care and hence would have high doses of drugs like Donepezil and Rivastigmine. Moreover population in the current study belongs predominantly to lower and middle socioeconomic class. Dose titrations of Donepezil and Rivastigmine were not reflected in this study because this study is a point prevalence study, so here patients were not serially evaluated but only assessed once. Moreover, the current study was not powered to study the doses in details, as that would have required a high number of patients which would have required a multicentre study. At the same time Rivastigmine happens to be more expensive as compared to Donepezil. Low prescribing of Galantamine can also be explained in similar lines. So considering the cost effectiveness issues in Indian clinical scenario this study favors more of Donepezil prescribing. Thus, here, we only intended to study the different category of drugs utilized.

Our study highlights that on an average each prescription had 4 ± 0.1 drugs prescribed in the retrospective population. On the other hand 5 ± 0.2 drugs were found to be prescribed in the prospective population as the mean number of drugs in a dementia patient's prescription. These results were found to be synchronous with results reported by Laroche et al. who report 6.3 ± 3.1 as an average number of drug in each prescription [21].

Regarding the antipsychotic drugs used for behavioral symptoms, results of our study conclude on an average in our study within the geriatric group population (85 prescriptions) that 0.72 ± 0.2 antipsychotic drug per prescription was prescribed. On contrary higher number of antipsychotic drug prescribing is noted in the female population whose age is below 65 years (2 drugs per prescription) as compared to male population below 65 years of age. Langballe EM in his study found out after studying a total of 33,816 individuals who received antidementia drugs that total concomitant use of antidementia drugs with psychotropic drugs was 57.4% in men and 65.8% in women. Concomitant use of antipsychotics with antidementia drugs was about 16% for both male and female patients. Of the total sample, 11.9% of the women and 11.7% of the men used acetyl cholinesterase inhibitor (AChEI) antidementia drugs concomitantly with an interacting psychotropic drug [22].

Our study found that Quetiapine, an atypical antipsychotic, was the most common antipsychotic used for behavioral and psychotic symptoms in 38.46% of cases in a dose range of 25 mg to 100 mg per day. In study reported by Prasad et al. [6] likewise the commonest antipsychotic used was Quetiapine, which was used in 24 patients (47%) in a dose range of 25 to 300 mg/day. The drug might be a choice since it has lesser propensity of causing extrapyramidal side effects as compared to the other antipsychotic drugs. Sedation seen with this drug could also be one of the confounding reasons for having to prescribe this drug. On the other hand Clozapine was prescribed in 9 patients that is, 11.53% of cases. In a study conducted by Tariot et al. [23] Clozapine has shown some efficacy in controlling psychosis and behavior disturbances in patients with Alzheimer's disease. Regarding Olanzapine, the greater metabolic side effects of this drug like weight gain, impaired glucose tolerance, and worsening of diabetes might have discouraged its use and, thus, reasons for its less prescribing. While Haloperidol, a drug belonging to class of older typical antipsychotic, although being potent antipsychotic was found to be prescribed in 3 prescriptions only. The second most prescribed drug was found to be a Benzodiazepine Lorazapam, Nitrazepam, and Nonbenzodiazepine hypnotics like Zolpidem probably due to their fewer side effects and cost effectiveness. On the contrary a cohort study conducted by Martinez et al. on trends in the prevalence of antipsychotic drug use among patients with Alzheimer's disease and other dementias including those treated with antidementia drugs in the community in the UK which enrolled 50 349 patients with incident dementia diagnosis and 50 349 matched controls, 10 794 first time users of AChEI and 669 of Memantine. The mean prevalence of antipsychotic use from 1995 to 2011 on diagnosis of dementia was 12.5%, decreasing from 19.9% in 1995 to 7.4% in 2011. There was an increase in antidepressant use (10.7–26.3%) and a small increase in anxiolytic use. This reduction is to be welcomed given the concerns about the overprescription of antipsychotics the risks of their use versus their limited benefits [24]. Even though studies suggest that there is an overall decrease in the prescribing of antipsychotics to dementia patients one can still observe high irrational use of this medication for the patients of dementia. While antipsychotics should be considered for patients suffering from dementia along with behavioral and psychotic symptoms, they should be prescribed only if specifically required or if other treatments have failed. Initial interventions for behavior disturbances should include cognitive, environmental, and social techniques. Many demented patients with behavior disturbances will not need psychotropic medication but can be managed successfully with nonpharmacologic techniques, such as the use of familiar objects, maintenance of sleep-wake cycles, redirection, and frequent reorienting (verbally or by posting a calendar in their room).

Results of the Phase 1 outcomes from the clinical antipsychotics Trial of Intervention Effectiveness study for Alzheimer's disease (CATIE-AD) suggest that antipsychotics may be more effective for particular symptoms like anger, agitation, aggression, and paranoid ideas. They do not appear to improve the functioning, care needs or quality of life [25]. The Food and Drug analysis of 17 double blind randomized placebo controlled trials among elderly people with dementia, commented that antipsychotics were associated with a significantly higher 1.6-1.7 times greater mortality risk when compared with placebos [26]. However there is a paucity of any evidence based treatment and delayed initiation of antipsychotics treatment.

Detailed analysis of the prescriptions under the current study reveals that there is high prescribing of vitamin B9, that is, folic acid and vitamin B12 in the patients without performing blood levels of the same and to find out the need for the same in patient. This is a judicious point to focus upon since literature supporting the use and benefits of same in dementia is still controversial. A study by Ford et al. reported that daily supplementation of vitamins B12, B6, and folic acid does not benefit cognitive function in older men, nor does it reduce the risk of cognitive impairment or dementia [27]. This is a judicious point to focus upon since literature supporting the use and benefits of same in dementia are still controversial. According to consensus guidelines on treatment of dementia before selecting a supplementation strategy, it is recommended that clear goals and target populations be set and, checking of vitamin B12 and folic acid levels should be included in the diagnostic workup in patients undergoing assessment for dementia [7]. In light of this in current practice there seems to be scarcity of data on prior determination of serum vitamin B12 and folic acid status by clinicians before supplementing these vitamins in patients of dementia. Over use of such multivitamins, and folic acid along with Anti dementia medications, without checking the serum levels may lead to an over prescribing of these drugs as well as increased economic burden to the patients. However there seems to be a growing question over the use of vitamin B12, folic acid along with antidementia medications in cognitively impaired patients as, it is often considered by treating physicians that patients in developing countries are underprivileged, with malnutrition which leads to frequent prescription of antioxidants and multivitamins without any major available evidence of strong benefit. While many times patients cannot afford the cost of these investigations and serum tests, so the prescribing clinicians prescribe vitamin B12 as well as folic acid to such patients routinely. On the other hand studies have documented fundamental roles of Folates and vitamin B12, in central nervous system function at all ages, especially in purine, thymidine, neucleotide, and DNA synthesis, genomic and nongenomic methylation and, therefore, in tissue growth, differentiation and repair. There is interest in the potential role of both vitamins in the prevention of disorders of central nervous system development, mood, dementia, including Alzheimer's disease and aging [28].

*Limitations.* In a developing country like India, dementia still remains as an under recognized public health burden. Although our study depicts a picture of drugs utilized in dementia, it strengthens the point that further long term research or twelve months long follow up or a multicentric trial should be conducted in a large population of dementia patients in this region. One can find prognosis in such patients and correlations between the treatment and MMSE scores can be ascertained. While one can serially evaluate patients by dose titration of medicines, one can also find out the adverse drug effects and cost effectiveness of the treatments given in such patients. However it is still unclear whether these results can be generalized to other populations without universal health coverage or drug formulary benefits.

Comorbidities in these patients were effectively managed with antihypertensive drugs and antidiabetic drugs and moreover we did not intend to study details of various drugs prescribed in varied comorbid condition in this study.

## 5. Conclusion

Keeping in mind the limitations of the current study, nonetheless this investigation has thrown up valuable results that will surely help fine tune the clinical prescription approach in dementia patients. Alzheimer's disease as other lifelong diseases needs to be more understood and analyzed in terms of its prescribing since it can have a major socioeconomic impact on its patients and caregivers particularly in developing countries like India. The audit highlights the need to study drug utilization pattern in dementia patients residing in developing countries, in terms of both pharmacovigilance and pharmacoeconomics and also strengthens the need to evaluate the rational use of vitamins and folic acid in these patients.

## Figures and Tables

**Table 1:** Depiction of different demographic parameters of study population.

Distribution parameter	No. of patients (*N* = 125)	
	Retrospective data	Prospective data
Gender distribution		
Male	51 (58.61)	24 (63.15%)
Female	36 (41.32%)	14 (36.82%)
Age range	45–94 years	52–93 years
Average age	70 ± 1.01 years	72 ± 2.01 years
Age distribution		
Below 65 years	28 (32.18)	12 (31.57%)
Above 65 years	59 (67.81%)	26 (68.42%)

Total no. of patients	87	38

**Table 2:** Socioeconomic classification of dementia cases.

SR. no.	Socioeconomic class	Retrospective data	Prospective data
1	Low	53	21
2	Middle	23	9
3	High	11	8

	Total	87	38

**Table 3:** Education status of dementia patients.

SR. no.	Patient education	Retrospective data	Prospective data
1	Illiterate	8	2
2	High school graduate	53	17
3	Graduate	20	10
4	Post graduate	6	9

	Total	87	38

**Table 4:** Depiction of subtypes of dementia.

SR. no.	Type of dementia	No. of patients
1	Lewy body dementia	2.4% (3)
2	Alzheimer's disease	65.6% (82)
3	Vascular dementia	21.6% (27)
4	Frontotemporal dementia	10.4% (13)

	Total	125

**Table 5:** Depiction of dose distribution of different antidementia drugs used.

SR. no.	Drug prescribed		Percentage
1	Donepezil	Percentage	58.4
	5 mg	68.49	
	10 mg	31.50	
	Total	**100**	

2	Rivastigmine	Percentage	13.63
	1.5 mg	41.17	
	3 mg	58.82	
	Total	**100**	

3	Donepezil (5 mg) + mementine (5 mg)		6.43

4	Galantamine (8 mg)		12.83

5	No. antidementia drug		8
